# Special Issue on “Advances in Skin Lesion Image Analysis Using Machine Learning Approaches”

**DOI:** 10.3390/diagnostics12081928

**Published:** 2022-08-10

**Authors:** Amirreza Mahbod, Isabella Ellinger

**Affiliations:** Institute for Pathophysiology and Allergy Research, Medical University of Vienna, 1090 Vienna, Austria

## 1. Introduction

Skin diseases are widespread and a frequent occurrence in general practice. Providing access to medical care and high accuracy of diagnosis are important issues, specifically when it comes to distinguishing benign conditions from malignancies such as melanomas where early diagnosis and treatment increase overall survival and cure rates [1].

Dermatologists diagnose skin lesions from dermoscopic or clinical images by performing visual inspection. To support the diagnostic process that might be slowed by an increasing workload as well as a lack of specialists in certain areas of the world, methods for computer-aided detection and computer-aided diagnosis for skin lesion image analysis have been developed [2]. Among them, advanced machine learning and especially deep learning (DL) approaches have reached dermatologist-level classification of skin lesions from dermoscopic and non-dermoscopic images [3]. Suitable cloud-based or offline computational resources and large publicly available databases for skin lesion images have promoted the development of dermatological applications of DL-based technologies for image analysis. Before their practical use in clinical settings, however, several issues need to be addressed, such as the generation of unbiased image datasets, the generalisability of models, the applicability of algorithms in real-world settings or transparency of the decision process of DL algorithms, to name a few.

This Special Issue presents the latest advances in the use of machine learning techniques for image analysis of skin lesions. A total of six papers were selected for publication, including one review article and five papers with excellent technical contributions. All articles underwent a peer-review process with typically two rounds of review, with at least three experts in the field carefully reviewing each submitted paper.

## 2. Papers Included in the Special Issue

In their study titled “Uncovering and Correcting Shortcut Learning in Machine Learning Models for Skin Cancer Diagnosis”, Nauta et al. [4] investigate the undesirable hidden errors and shortcut learning in training a black-box DL-based model for skin lesion classification. Specifically, they show how the presence of colour calibration charts in part of the ISIC dermoscopic skin lesion images led to unreliable predictions of the VGG-16 model. The results confirm that shortcut learning can be largely removed from a skin lesion classification model by proposing a model agnostic method based on the technique of image inpainting to insert colour calibration charts into all skin lesions images. The study shows that DL-based model performance for skin lesions image analysis should be evaluated with greater caution.

In their study titled “Integrating Domain Knowledge into Deep Learning for Skin Lesion Risk Prioritization to Support Referral in Teledermatology”, Carvalho et al. [5] present a novel hybrid DL-based method for skin lesion risk prioritisation. The proposed pipeline consists of image preprocessing, lesion segmentation, various training schemes for skin lesion classification, and a separate convolutional neural network (CNN)-based model for prioritisation. In a final fusion scheme, the results of the skin lesion classification model, the prioritisation model, and the hand-crafted prior knowledge maps are combined to classify images into three classes (normal, priority, and high priority). When applied to a dataset from the Portuguese National Health System, the authors obtained excellent classification results and demonstrated a practical example of using prioritisation to assist teledermatology referral.

Chen et al. [6], in their work entitled “Computer-Aided Detection (CADe) System with Optical Coherent Tomography for Melanin Morphology Quantification in Melasma Patients”, developed an automatic method for quantifying melanin in full-field optical coherence tomography (FF-OCT) images. The proposed method consists of several components, including a spatial compounding-based denoising CNN, a contrast enhancement module, an object segmentation module, and a post-processing module to derive melanin features from melasma and perilesional skin. Experimental results based on cheek images of eight Asian patients show significant differences between the lesion and perilesional skin for a subset of the extracted features. The proposed algorithms can be used as an objective tool to study the clinically relevant melanin features in FF-OCT images.

Moldovanu et al. [7], in their study entitled “Towards Accurate Diagnosis of Skin Lesions Using Feedforward Back Propagation Neural Networks”, investigate the effect of node number and asymmetry features on the skin lesion classification performance of a conventional artificial neural network (ANN). By exploring sixty different architectures, the experimental results showed that using sixteen nodes in a three-layer ANN and A2 asymmetry feature yielded the best overall classification performance. While the proposed approach did not use any sophisticated features and architectures compared to advanced CNN-based models, the derived results were thoroughly interpretable.

Khan et al. [8], in their work titled “Skin Lesion Segmentation and Multiclass Classification Using Deep Learning Features and Improved Moth Flame Optimization”, present a multi-step pipeline for segmentation and classification of dermoscopic images. The proposed model consists of a colour enhancement module, a deep saliency segmentation module, a module to extract lesion features using pre-trained CNNs, an improved moth lame optimisation method to select the best features, and finally, a kernel extreme learning machine as a lesion classifier. When applied to four publicly available datasets, excellent classification and segmentation results were obtained compared to several state-of-the-art algorithms.

Kassem et al. [9] provide a comprehensive review of studies on automated image analysis of skin lesions in their paper entitled “Machine Learning and Deep Learning Methods for Skin Lesion Classification and Diagnosis: A Systematic Review”. In this work, the authors discuss the strengths and bottlenecks of methods from 102 machine learning and DL-based articles published over the past five years. The authors also point out important research directions for future studies in the field of skin lesion classification.

## 3. Conclusions

In this editorial, we briefly introduced the promising articles selected for this Special Issue and hope that they will inspire interested researchers to pursue future studies. Finally, we would like to thank all the authors who submitted their valuable manuscripts and the *Diagnostics* support team for their help in preparing and finalizing this Special Issue. We would also like to express our gratitude to all reviewers for their timely and professional comments.

## Data Availability

Not applicable.
